# Prospective study to evaluate radioactive iodine of 20 mCi vs 10–15 mCi in Graves’ disease

**DOI:** 10.1186/s12902-024-01588-3

**Published:** 2024-04-25

**Authors:** Wasit Kanokwongnuwat, Nawarat Penpong

**Affiliations:** 1 Division of Nuclear Medicine, Department of Radiology, Phrapokklao Hospital, No 38, Leab Neon Road, Mueang, Chanthaburi, 22000 Thailand; 2 Division of Endocrinology, Department of Internal Medicine, Phrapokklao Hospital, No 38, Leab Neon Road, Mueang, Chanthaburi, 22000 Thailand

**Keywords:** Graves' disease, Radioactive iodine, Hyperthyroidism, Increasing dose, Treatment efficacy, Remission

## Abstract

**Objectives:**

To assess whether increasing radioactive iodine dose can increase treatment efficacy in Graves’ disease.

**Methods:**

A prospective study was conducted, including 106 patients receiving 20 mCi (740 MBq) radioactive iodine (RAI), compared with a retrospective data, including 113 patients receiving 10–15 mCi (370–555 MBq) RAI. Remission and failure rates were evaluated at 6 months post-RAI. Statistical analysis was performed using logistic regression and Kaplan–Meier curves.

**Results:**

Patients receiving 20 mCi RAI demonstrated a significantly higher remission rate compared to the 10–15 mCi group (82.1% vs 66.4%, *p* = 0.009). Median time to remission was shorter in the 20 mCI group (3 vs 4 months, *p* = 0.002). Hypothyroidism at 6 months was more prevalent in the 20 mCi group (67% vs 53%, *p* = 0.03). Larger thyroid size (> 60 g) was associated with treatment failure (*p* = 0.02).

**Conclusions:**

Higher dosage (20 mCi) RAI showed superior efficacy in achieving remission compared to lower dosages (10–15 mCi) in Graves’ disease treatment.

## Background

Graves’ disease (GD) is the most common cause of hyperthyroidism [1]. It is an autoimmune disease and presents a significant challenge in clinical management due to its diverse manifestations and potential complications [2]. Radioactive iodine (RAI) therapy stands as a cornerstone in the treatment options, offering a targeted approach to cure thyroid overactivity. The goal is to provide sufficient radiation dose to make the patient hypothyroid. This result can be done by a fixed dose method or a calculating dose method with the same effectiveness [3]. Both cannot guarantee remission, but the calculating one requires additional time and computation. Therefore, many institutions have stopped using the calculating method and prefer the fixed dose method. The optimal dosage of RAI for achieving remission is recommended with 10–15 mCi (370–555 MBq) [4] which shows a remission rate of 90% [5–8]. However, there are some reports regarding a lower remission rate of about 50–80% [9–12]. Correlation between the remission after RAI therapy and administered radiation dose is seen in some studies [6, 13–16]. This prospective study aims to address the benefit of increasing radiation dose by comparing the efficacy of two RAI dosages: 20 mCi (740 MBq) vs 10–15 mCi (370–555 MBq).

## Methods

### Subjects

We used retrospective data, including 113 patients with GD who received RAI 10–15 mCi in the previous year between April 2021 – June 2022 at the Phrapokklao Hospital in Thailand as a comparison. Eligible patients were diagnosed with GD based on clinical presentation and laboratory findings and aged 18 or more. The exclusion criteria included previous RAI treatment or thyroidectomy, active or severe ophthalmopathy, pregnancy or lactation, and co-existing thyroid cancer. The prospective group included 106 patients between July 2022 – June 2023, who received RAI 20 mCi at the same institution.

### Data collection

Clinical and laboratory data were collected before the treatment for 1–2 months and after the treatment every 1–2 months until 6 months. The goiter size was assessed 1–2 months before the RAI by palpation. If value discrepancy were detected by different physicians, the average size was recorded. Thyroid scan or US was done when a thyroid nodule was palpated. The severity of Graves’ orbitopathy (GO) and clinical activity score to determine active GO were evaluated before the RAI. All clinical examinations were done by physicians including general practitioners, endocrinologists, internists and nuclear medicine physicians. The referral to ophthalmologist was done in case of severe or active orbitopathy. TSH, free T3 and free T3 levels were measured by chemiluminescent microparticle immunoassay (Anility i). The reference range for laboratory values were TSH 0.35–4.94 uIU/mL, free T3 2–4.4 pg/mL, free T4 0.93–1.71 ng/dL. TSH receptor antibody was rarely measured due to long turn-around time and high cost.

### Protocol for RAI therapy

All patients were advised to stop antithyroid drugs (ATD) and take low iodine diet for 7 days before RAI. RAI was administered with a fixed dose basis of 10–15 mCi for the retrospective group and 20 mCi for the prospective group. Thyroid gland size and thyroid uptake were not used to determine the RAI dose. Thyroid uptake was not measured in this study due to increased hospital visits and clinical non-significance. ATD and regular diet were resumed 3 days after RAI.

### Definition of remission and failure

The outcomes were measured at 6 months after RAI. Remission was defined as euthyroid without ATD for a month, hypothyroid or thyroxin requirement. Failure was defined as persistent hyperthyroid or unable to withdraw ATD. The study was approved by the Chanthaburi Research Ethics Committee (COA032/65). All experimental protocols were approved by Chanthaburi Research Ethics Committee. Informed consents were obtained from all subjects and/or their legal guardian(s).

### Statistical analysis

The baseline characteristics of the two groups were compared using chi-square or Fisher’s exact test for qualitative data and t-test or Mann Whitney U test for quantitative data. We used logistic regression analysis to evaluate factors associated with treatment failure and Kaplan–Meier curves with log-rank test to compare cumulative remission rates of the two groups. A two-sided *p*-value of less than 0.05 was considered statistically significant. The data were analyzed using IBM SPSS Statistics (Version 29).

## Results

Baseline characteristics were similar except for the lower TSH and higher Free T3 in the 10–15 mCi group (Table 1). Most patients used methimazole (MMI) before RAI. There were 3 patients in the 20 mCi group who used propylthiouracil (PTU) instead of MMI before the RAI. The PTU doses were converted to MMI by divided by 10 [17]. One patient did not receive ATD before the RAI. Ophthalmopathy was seen in 15 patients in the 10–15 mCi group and 13 patients in the 20 mCi group. These patients had mild degree and inactive disease. In the 10–15 mCi group, 18 patients received RAI 10 mCi and 95 patients received RAI 15 mCi. There were 3 patients who had thyroid nodule They underwent thyroid scans and revealed diffuse thyroid uptake with a cold nodule. Fine needle aspirations were done and proved to be benign. Two patients got tested with TSH receptor antibody and reported high level.

**Table 1:** Baseline characteristics of patients with Graves’ disease, according to radioactive iodine dose administration 10–15 mCi vs 20 mCi

	RAI 10–15 mCi (*N* = 113)	RAI 20 mCi (*N* = 106)	P
Age (year)	44.8 ± 13.5	45 ± 15.5	0.98
Sex (female/male)	81/32	79/27	0.64
BMI (kg/m2)	24 ± 4.9	24.5 ± 5	0.78
MMI dose (mg)	7.5 (2.5–10)	10 (5–15)	0.15
MMI duration (months)	28 (8.5–49.5)	25 (6.8–39.8)	0.41
Ophthalmopathy (n)	15/113	13/106	0.82
Thyroid size (grams)	54.9 ± 20.4	49.2 ± 24.2	0.23
TSH (uIU/mL)	0.006 (0.005–0.032)	0.01 (0.005–1.459)	0.007*
Free T3 (ng/dL)	4.9 (3.5–9.6)	4.2 (3.2–6.1)	0.037*
Free T4 (nd/dL)	1.8 (1.3–2.7)	1.4 (1–2.2)	0.12

The values are mean ± SD or median (quartile 1st-3rd)

*RAI* Radioactive iodine, *BMI* Body mass index, *MMI* Methimazole

* statistical significance

**Fig. 1:**
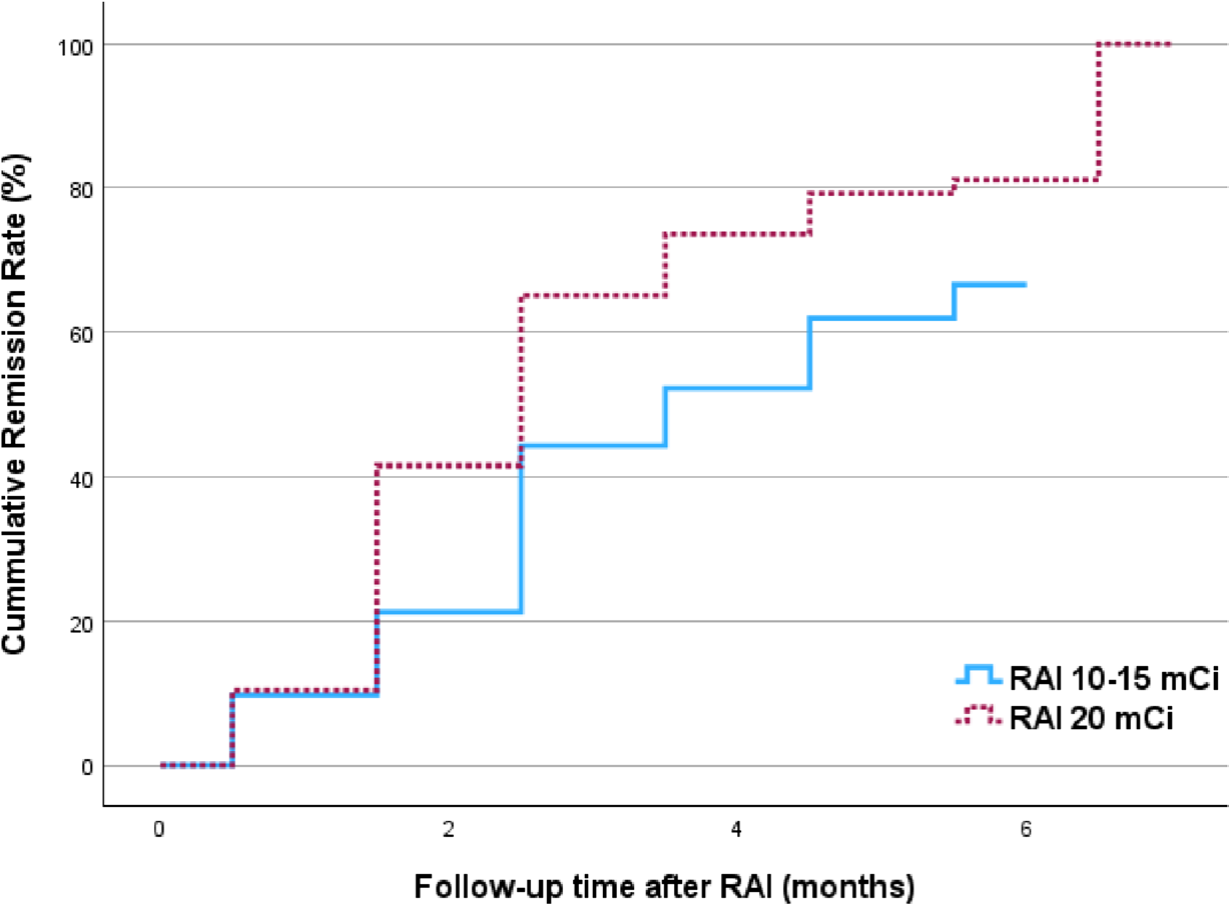
Kaplan–Meier curves showing remission rates between radioactive iodine 10–15 mCi vs 20 mCi. RAI, radioactive iodine

The remission rate was higher in the patients receiving RAI 20 mCi compared to 10–15 mCi (82.1% vs 66.4%, *p* = 0.009). The Kaplan–Meier curves showed the median time to remission was shorter in the 20 mCi group (3 months, range 2.6–3.4 months vs 4 months, range 3–5 months, *p* = 0.002) (Fig. 1). At 6 months, hypothyroid presented more in the 20 mCi group (67% vs 53%, *p* = 0.03).

Thyroid size was a significant predictor for a treatment failure (*p* = 0.002) (Table 2). There was no correlation among other clinical features. The thyroid size cutoff more than 60 g was associated with RAI treatment failure (*p* = 0.02).

**Table 2:** Factors associated with radioactive iodine treatment failure using logistic regression analysis

	Remission	Failure	P
Age (year)	45.3 ± 14.8	43.8 ± 13.5	0.49
Sex (female/male)	122/40	38/19	0.2
BMI (kg/m2)	24.5 ± 5.1	23.6 ± 4.3	0.26
MMI dose (mg)	7.5 (5–10)	10 (2.5–15)	0.74
MMI duration (months)	27 (7.8–42.2)	25 (6.5–60)	0.22
Ophthalmopathy (n)	17/162	11/57	0.09
Thyroid size (grams)	49.4 ± 21.9	60.1 ± 22.3	0.002*
TSH (uIU/mL)	0.008 (0.005–0.16)	0.008 (0.005–0.9)	0.35
Free T3 (ng/dL)	4.7 (3.4–8.7)	4.5 (2.9–8.2)	0.71
Free T4 (nd/dL)	1.7 (1.2–2.6)	1.5 (1–2.3)	0.66

The values are mean ± SD or median (quartile 1st-3rd)

*RAI* Radioactive iodine, *BMI* Body mass index, *MMI* Methimazole

* statistical significance

Subgroup analysis of patients with thyroid gland size 60 g or less compared to more than 60 g (Table 3). Patients with thyroid gland size with 60 g or less had better response to RAI. Although, with larger gland size, RAI 20 mCi had better remission rate but not statistically significant.

**Table 3:** Subgroup analysis of patients with thyroid gland size ≤ 60 g vs > 60 g

	Remission	Failure	P
Overall	(*N* = 162)	(*N* = 57)	
RAI 10–15 mCi	75 (66.4%)	38 (33.6%)	0.009*
RAI 20 mCi	87 (82.1%)	19 (17.9%)	
Thyroid size ≤ 60 g	(*N* = 86)	(*N* = 20)	
RAI 10–15 mCi	32 (69.6%)	14 (30.4%)	0.011*
RAI 20 mCi	54 (90%)	6 (10%)	
Thyroid size > 60 g	(*N* = 76)	(*N* = 37)	
RAI 10–15 mCi	43 (64.2%)	24 (35.8%)	0.4
RAI 20 mCi	33 (71.7%)	13 (28.3%)	

*RAI* Radioactive iodine

* statistical significance

We did not see any progression or new development of ophthalmopathy. However, we observed some adverse events between both groups (Table 4). All events were mild and resolved without hospitalization. No differences in any events for one patient in 10–15 mCi vs 20 mCi group (32.7% vs 40.6%, *p* = 0.2). Nevertheless, there were trends of adverse events toward the 20 mCi in nausea and vomiting, neck tenderness, and sorethroat.

**Table 4:** Adverse events, number of patients (%)

Event	RAI 10–15 mCi (*N* = 113)	RAI 20 mCi (*N* = 106)	P
Any adverse events for one patient	37	43	0.2
Dizziness	4	3	1
Dyspnea	14	11	0.6
Fatigue	4	4	1
Fever	1	2	0.6
Insomnia	2	0	0.5
Muscle cramps	0	4	0.053
Nausea and vomiting	2	10	0.01*
Neck tenderness	0	5	0.03*
Palpitation	15	8	0.2
Rash	0	2	0.3
Sorethroat	0	5	0.03*
Sweating	1	2	0.6

*RAI* Radioactive iodine

* statistical significance

## Discussion

Our findings indicate that the higher RAI dosage of 20 mCi led to a significantly higher remission rate compared to the lower dosage range of 10–15 mCi. This result aligns with previous studies suggesting a correlation between administered radiation dose and treatment outcomes [15]. Some studies showed no difference in increasing radiation doses which could be caused by low statistical power [5, 11]. In some circumstances, lower remission of 50–80% [9, 12, 15] might not be acceptable for patients and physicians. This lower remission rate could be explained by iodine repletion status in Thailand [18] and other countries [15, 19]. The iodine consumption of Thai adults is 11.5 g for adults, which is far higher than the recommendation by World Health Organization (WHO) of 150 mcg/d [20].

Importantly, the median time to remission was shorter in the 20 mCi group in our study, suggesting a more rapid onset of therapeutic effect with the higher dosage. This finding has potential implications for patient management, as a shorter time to remission may translate to faster resolution of symptoms and a quicker return to euthyroid status. Additionally, the higher proportion of patients presenting with hypothyroidism at 6 months in the 20 mCi group highlights the effectiveness of this dosage in achieving the desired therapeutic outcome. However, the smaller gland size of the 20 mCi group could affect the remission rate (*p* = 0.023).

Thyroid size appeared as a significant predictor of treatment failure, with larger thyroid glands (> 60 g) associated with a higher risk of failure. This outcome aligns with the previous literatures [8–10, 21, 22]. We did not find other significant factors previously mentioned such as male sex and free T4 [8, 23]. Thyroid with a smaller gland size seems to response well to RAI and even better with higher radiation dose, unfortunately with gland size more than 60 g, the effectiveness did not improve in this study. This finding emphasizes the importance of considering individual patient characteristics in treatment decision-making and dosage selection. Future studies could explore additional factors contributing to treatment response and failure to further optimize patient outcomes.

In terms of safety, we observed mild adverse events in both dosage groups, with no significant differences between the 10–15 mCi and 20 mCi groups. These findings suggest that the higher dosage of 20 mCi was well-tolerated and did not result in a disproportionate increase in adverse events. However, in some adverse events, we observed more in the RAI 20 mCi group which could be explained by worsened hyperthyroid after receiving the RAI [24, 25]. All events were mild and resolved without hospitalization. Nevertheless, continued monitoring of adverse events and long-term outcomes is warranted to ensure the safety of RAI therapy in patients with GD.

There are some concerns about cancer risk regarding RAI treatment. The data from the Cooperative Thyrotoxicosis Therapy Follow-up Study (CTTFUS) showed no difference of the solid cancer standard mortality ratio among patients treated with radioactive iodine, surgery, or antithyroid drugs, although, there was a dose response relationship between RAI dose and solid cancer mortality [26, 27]. Minimal risk of malignancy was also observed in other studies [28, 29]. The counseling for RAI seems reasonable to neglect small cancer risk, as compared to antithyroid drugs or surgery [30]. We think that increasing radiation dose for more treatment efficacy might improve patients’ overall health and reduce the risk of receiving additional radiation, in case of treatment failure.

Limitations of our study include its non-randomized and non-double blinded nature and potential confounding factors that were not accounted for in the analysis. Additionally, the relatively short follow-up period of 6 months may not capture long-term treatment outcomes and recurrence rates, however, these 6 months period is practical in our practice [4]. Future research could address these limitations by conducting randomized controlled trials with longer follow-up periods and comprehensive outcome assessments.

In conclusion, this prospective study highlights the efficacy of RAI 20 mCi in GD treatment, offering faster remission and higher success rates compared to lower dosages. Individualized treatment based on thyroid size is crucial for optimizing outcomes in GD patients.

## Data Availability

Dataset used in this study is available upon reasonable request to the corresponding author via wasit.k@chula.ac.th.
